# The Effect of CoAl_2_O_4_ as a Nucleating Agent and Pouring Temperature on the Microstructure and Properties of Inconel 713C^®^ Nickel-Based Superalloy Castings

**DOI:** 10.3390/ma16165588

**Published:** 2023-08-11

**Authors:** Rafał Cygan, Łukasz Rakoczy

**Affiliations:** 1 Consolidated Precision Products Corporation, Investment Casting Division, Hetmańska 120, 35-078 Rzeszow, Poland; 2 Faculty of Metals Engineering and Industrial Computer Science, AGH University of Krakow, Mickiewicza 30, 30-059 Krakow, Poland; lrakoczy@agh.edu.pl

**Keywords:** superalloy, inoculant, Inconel, shell mold, aerospace

## Abstract

In this work, three melt-pouring temperatures (1450 °C, 1480 °C, 1520 °C) and CoAl_2_O_4_ inoculant contents in the shell mold’s primary coating (0 wt%, 5 wt%, and 10 wt%) were selected to study microstructural and mechanical property changes of the Inconel 713C^®^ nickel-based superalloy. The castings’ phase transformation temperatures, phase constitution, microstructure, and mechanical properties at room and elevated temperatures were investigated via thermodynamical simulations, differential thermal analysis, light and scanning electron microscopy, energy-dispersive X-ray spectroscopy, and tensile and stress-rupture tests. The pouring temperature and inoculant content strongly influenced the mean equiaxed grain size, which ranged between 2.36 and 6.55 mm. The primary microstructure of Inconel 713C^®^ castings, owing to its complex chemical composition, comprised multiple phases, including γ, γ’, MC, M_3_B_2_, and Ni_7_Zr_2_. The mean size of γ’ was in the 0.446–0.613 μm range, depending on the casting variant. Grain refinement with CoAl_2_O_4_ at ambient temperature for each melt-pouring temperature led to increased yield strength (YS) and ultimate tensile strength (UTS). YS was in the range of 775–835 MPa, while UTS was in the range of 868–1010 MPa. A reverse trend was observed in samples that crept in 982 °C/152 MPa, while for each variant, the time to rupture exceeded 30 h. The maximum time to rupture was 46.1 h obtained in the unmodified casting poured at 1480 °C.

## 1. Introduction

Inconel 713C^®^ is classified as a nickel-based superalloy known for its exceptional properties at elevated temperatures [1]. It exhibits high mechanical strength and excellent resistance to oxidation and hot corrosion, making it suitable for manufacturing components for aerospace engines and industrial gas turbines [2]. Several physical factors contribute to the usefulness of Ni-based superalloys in harsh service environments. The face-centered cubic (FCC) structure of the γ matrix is characterized by low rates of thermally activated processes, resulting in lower creep deformation. The FCC crystal structure also remains thermodynamically stable from the temperature of liquid nitrogen to the melting point. The γ matrix is strengthened by coherent γ’ precipitates, characterized by an ordered L1_2_ structure [3]. This leads to a yield stress anomaly, where the flow stress increases with temperature, making it advantageous for high-temperature components [4]. Due to the high costs associated with superalloy machining, investment casting is crucial for fabricating elements with complex geometries [5]. The lost-wax process is particularly effective, offering high dimensional accuracy by using monolithic ceramic molds [6]. The cost difference between investment casting and other casting methods arises from the need to create disposable patterns and construct individual molds. Investment casting of Inconel 713C^®^ primarily focuses on producing blades and vane clusters, typically for low-pressure turbine (LPT) sections [7]. Over the years, advancements in the complexity and integrity of lost-wax castings have led to continuous improvements in the manufacturing process. The superior mechanical properties of nickel-based superalloys mainly stem from the alloying elements found in their matrix, casting parameters, and heat treatment consisting of solution treatment and aging [8]. Notably, sufficiently high resistance to creep and low-cycle fatigue of the Inconel 713C^®^ alloy can be achieved in as-cast conditions, eliminating the need for expensive heat treatment.

The continuous improvement of engines has made the LPT sections more structurally complex. Turbine components must possess an appropriate microstructure and be free of casting defects that could lead to catastrophic failure [9]. Operating temperatures can reach up to 700 °C, necessitating a fine-grained microstructure with high resistance to low-cycle fatigue (LCF) [10] and sufficiently good creep resistance [11]. Cast aerospace engine components produced by conventional lost-wax casting often have a coarse and inhomogeneous grain structure. However, these features can be controlled by adjusting the melt-pouring temperature and the composition of the prime coating in the shell mold [12]. Increasing the pouring temperature can enhance the filling ability of thin-walled blades and vanes but can also lead to grain coarsening due to decreased cooling rates [13]. The operating temperature in the LPT section is typically below the intensive creep range [14]. To address this issue, castings generally are modified by adding refiners, which contain highly stable particles, or by introducing an inoculant to the prime coat of the ceramic mold [15,16]. Grain refinement in the primary microstructure is directly related to increased heterogeneous nucleation sites during solidification [17]. Introducing inoculants can make the microstructure more uniform. Cobalt aluminate (CoAl_2_O_4_) is the most frequently utilized inoculant in the lost-wax casting process of nickel-based superalloys [18]. This compound is one of the representatives of complex oxides with a common formula AB_2_O_4_ in which “A” ions are divalent cations filling tetrahedral sites and “B” ions are trivalent cations in octahedral sites [19]. Cobalt aluminate is usually fabricated by firing cobalt (II, III) oxide (CoO·Co_2_O_3_) and aluminum hydroxide (Al(OH)_3_) at 1200–1300 °C. The solid-state reaction between cobalt oxide and aluminum hydroxide results in the formation of the CoAl_2_O_4_ spinel-structured compound [20]. The amount of cobalt aluminate added to the primary slurry can vary, usually between 1.0 and 10.0 wt% or more, and is determined by the superalloy grade, casting geometry, airfoil thickness, and aerospace customer specification. Too high concentrations do not significantly influence grain size or, in turn, the mechanical properties. However, they may lead to higher manufacturing costs or the presence of non-metallic inclusions. With this in mind, the aim is to determine the influence of CoAl_2_O_4_ content in the prime coat and melt-pouring temperature on the macrostructure, microstructure, and mechanical properties of Inconel 713C^®^ superalloy at room and elevated temperatures.

## 2. Materials and Methods

**Figure 1:**
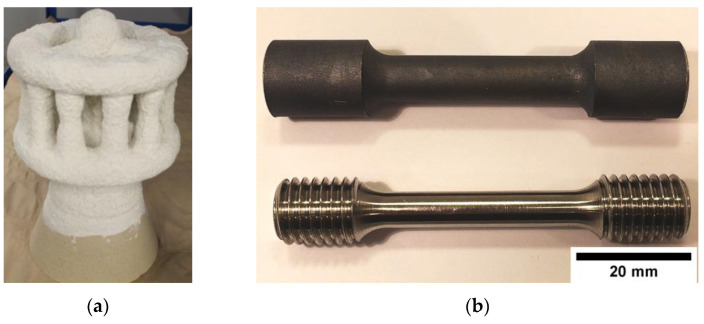
(**a**) Prepared shell mold; (**b**) casting geometry and machined sample for mechanical property testing.

The Inconel 713C^®^ nickel-based superalloy was used for the castings and gating systems. Its chemical composition obtained via spark optical emission spectroscopy was the following (wt%): Cr—14.24, Al—5.93, Mo—4.29, Nb—2.45, Ti—0.92, C—0.11, Zr—0.08, Co—0.04, B—0.012, Ni—Bal. The wax patterns were injection molded, and then monolithic ceramic molds were built up around these patterns through several dipping–drying–sieving cycles until the desired shell thickness was obtained. Alumina grit was applied as the primary stucco, while backup coats were made of alumina silicate powder and colloidal silica binder-based ceramic slurries, with alumina silicate grit serving as the backup stucco. Three different shell mold prime coatings were prepared: (a) zircon filler and colloidal silica binder (molds 1–3), (b) addition of 5 wt% of CoAl_2_O_4_ inoculant (molds 4–6), and (c) addition of 10 wt% of CoAl_2_O_4_ inoculant (molds 7–9). The wax was removed from the inside of the molds within a high-pressure boiler clave. Next, each mold was covered with a layer of alumina silicate Fiberfrax^®^ (UNIFRAX I LLC, Tonawanda, NY, USA) insulation. Pre-annealing was conducted at 600 °C for 2 h to strengthen the entire assembly and remove any remaining wax residue. The 4 kg ingots were melted using an induction furnace with zirconia crucibles in a VIM IC Consarc furnace under a 2 × 10^−3^ Torr vacuum. Each ceramic mold was placed in the heating chamber, preheated to 1150 °C, and held for 2 h. The liquid metal was poured at three temperatures T_0_ (monitored using Pt/Pt-Rh thermocouples): 1450 °C (L—low), 1480 °C (M—medium), and 1520 °C (H—high). After pouring, the mold was transferred from the heating zone to the cooling zone of the furnace within 10 s, followed by 60 s of ventilation in the cooling chamber. Once the castings had cooled to room temperature, the molds were removed and 9 casting variants were subjected to analysis (Table 1). The shell mold, single casting, and machined sample are presented in Figure 1.

Thermo-Calc^®^ software ver. 2022 (Thermo-Calc Software AB, Stockholm, Sweden) with the TCNI10 database was used to characterize the solidification path of Inconel 713C^®^ using the Scheil model. The model considers the segregation of solute elements during solidification and assumes the absence of back-diffusion during calculations. Complete solidification is assumed when less than 1% liquid is present in the calculation, and generally, the solidification range is quite broad [21]. Here, the model was used to predict the type of primary phases in the as-cast superalloy. The phases’ temperature stability between 700 and 1400 °C under equilibrium was also analyzed. Using a Netzsch STA 449F3 Jupiter (Netzsch, Selb, Germany) thermal analyzer equipped with a rhodium furnace operating up to 1600 °C (microbalance resolution 10^−6^ g, calorimetric sensitivity 0.1 mW), differential thermal analysis (DTA) was performed to obtain phase transformation temperatures during Inconel 713C^®^ superalloy cooling and heating. Prior to measurements, the device was calibrated, and the furnace’s vacuum chamber was filled with nitrogen. The samples were placed in Al_2_O_3_ crucibles and studied in the temperature range of 1000 °C to 1400 °C (20 °C/min).

For macro- and microscopic observation, the cross-sectioned samples were mounted in resin, metallographically prepared, and electrochemically etched in 10% oxalic reagent for 5 s. The commercial software ImageJ 1.53k (National Institutes of Health and the Laboratory for Optical and Computational Instrumentation, University of Wisconsin, 1.51j8, Madison, WI, USA) was used to quantitatively analyze the macrostructures imaged using a Leica i9 microscope (5 images per casting). The mean area, perimeter, and grain size (diameter of an equivalent circle) were calculated. The samples’ microstructures were imaged using a Leica DM light microscope (LM) and a Phenom XL (ThermoFisher Scientific, Lenexa, KS, USA) scanning electron microscope (SEM), equipped with an energy-dispersive X-ray (EDX) spectrometer (accelerating voltage was 15 kV). The obtained SEM backscattered electron (BSE) images (magnification ×20k) were subjected to binarization and de-spackling to remove noise without blurring any feature edges.

Considering that γ’ precipitates in superalloys usually take on a cubic-like morphology, their mean size was taken as the square side of the precipitates (square root of the precipitates’ area). Measurements were performed in 10 regions within the dendritic regions. Only γ’ precipitates characterized by an area within the 0.02–4.0 μm^2^ range were chosen.

Tensile testing was performed on an INSTRON 3382 (INSTRON Deutschland GmbH, Darmstadt, Germany) at room temperature, following the requirements of ASTM standard E8M-13a [22]. From there, the ultimate tensile strength (UTS), yield strength (YS), and elongation were estimated. Creep rupture tests were performed on a Walter + Bai AG LFMZ-30 (Walter + Bai AG, Löhningen, Switzerland) machine, in line with ASTM standard E139-11 [23]. The specimens (M12) were preheated to 982 °C, annealed for 1 h, and loaded with an axial force, which induced tensile stresses of 152 MPa in cross-sections.

## 3. Results

### 3.1. Analysis of the Solidification Path and Phase Stability in Inconel 713C^®^

**Figure 2:**
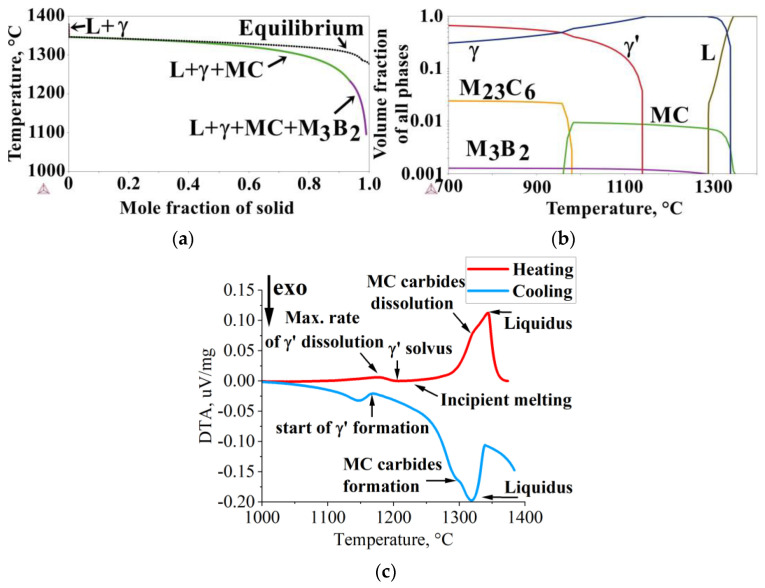
(**a**) Inconel 713C^®^ solidification path calculated via the Scheil model; (**b**) phase stability with increasing temperature under equilibrium conditions; (**c**) DTA curves registered during cooling and heating.

Based on the Inconel 713C^®^ nickel-based superalloy’s chemical composition, a thermodynamic simulation of the solidification path (Scheil method) was performed (Figure 2a). Superalloy crystallization (γ phase formation) began at 1382 °C. After reaching a γ phase fraction of 0.002 at 1347 °C, MC carbide precipitation from the liquid occurred, which is most favorable in interdendritic spaces (following typical reactions L→MC or L→γ + MC). The formation of MC carbides in the interdendritic liquid induces a depletion in strong carbide-formers, such as Nb and Ti [24]. With decreasing temperatures, the solubility of B in the matrix is reduced, inducing its segregation at the γ phase dendrite–interdendritic liquid interface and leading to M_3_B_2_ precipitation from the liquid at 1234 °C. According to the Scheil simulation, the equilibrium solidus temperature is 1276 °C. Secondary γ’ precipitates are not included in the results as they start precipitating in a solid state from a supersaturated matrix.

Under equilibrium conditions, the main strengthening phase is the γ’ phase, which dominates the structure up to 985 °C (Figure 2b). With further temperature increase, the γ takes over as the dominant phase. The solvus temperature of the γ’ phase is 1140 °C. The simulation indicates that M_23_C_6_ carbides are more stable than MC in the intermediate temperature range, suggesting the possibility of an MC + γ→γ’ + M_23_C_6_ phase transformation during service or heat treatment [25]. At high temperatures (exceeding 975 °C), MC carbides are more stable than M_23_C_6_ ones. The solvus temperature of M_23_C_6_ carbides is 980 °C. M_3_B_2_ borides are very stable up to the temperature of 1290 °C. After exceeding this temperature, they rapidly dissolve, with the possible appearance of a liquid phase caused by matrix enrichment in B. The liquidus temperature is 1340 °C, whereas MC carbides dissolve completely at 1348 °C.

DTA curves registered during specimen cooling and heating are shown in Figure 2c. During cooling, crystallization began at 1319 °C, followed by the precipitation of MC carbides at 1294 °C. Subsequently, a γ-γ’ eutectic and minor phases may form over a wide temperature range. A peak onset around 1168 °C suggests that after crystallization concludes, the formation of secondary γ’ precipitates from the γ solid solution commences. On the DTA heating curve, a peak is recorded at approximately 1176 °C, which probably corresponds to the maximum dissolution rate of γ’ intermetallic phase precipitates. Shortly after exceeding this temperature (at 1201 °C), the solvus temperature of γ’ is observed. Heating beyond 1244 °C causes sample melting, and the liquidus temperature was reached at 1344 °C.

### 3.2. Macro- and Microstructure of the as-Cast Inconel 713C^®^ Castings

**Figure 3:**
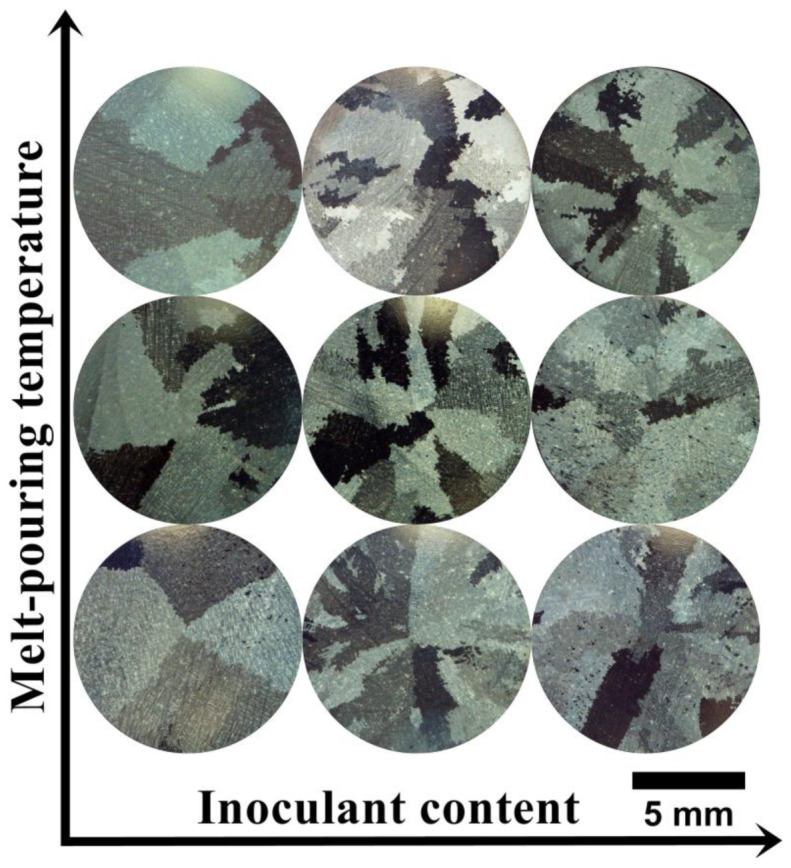
Macrostructure of the as-cast Inconel 713C^®^ castings.

The macrostructures of all casting variants are shown in Figure 3. Equiaxed grains of various sizes and distribution are visible. Casting defects, like chill zone structures, hot cracks, or misruns, were not observed. The relationship between surface grain size, melt-pouring temperature, and cobalt aluminate content in the primary coating is presented in Table 2. Increasing the CoAl_2_O_4_ content led to a decrease in grain size for each melt-pouring temperature. For the lowest temperature (1450 °C), the mean grain size changed from 4.40 mm to 2.44 mm when 10 wt% inoculant was applied. The greatest refining effect was obtained at T_0_ = 1520 °C, in which the grain size decreased from 6.55 mm (unmodified variant) to 2.36 mm (10 wt% of CoAl_2_O_4_). The relatively high grain size standard deviation values are due to the presence of both large and fine grains.

**Figure 4:**
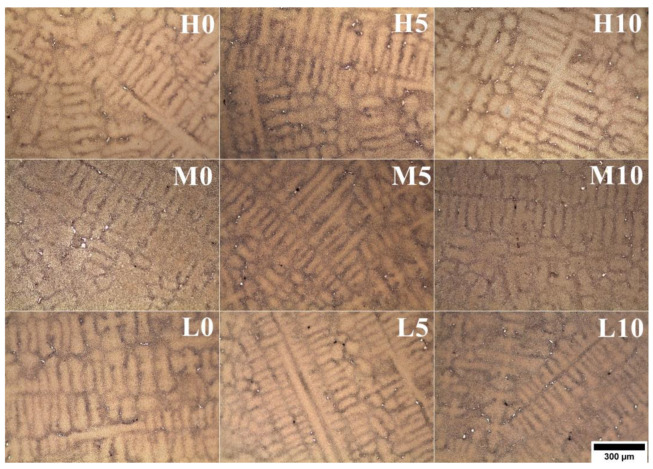
Dendritic microstructure of the as-cast Inconel 713C^®^ castings, LM.

The casting microstructures reveal typical dendritic structures, with features of the primary dendrite cores clearly visible (Figure 4). The dendritic areas (primary cores and secondary arms) are characterized by a relatively homogeneous microstructure. Within the interdendritic regions, γ-γ’ eutectic islands and fine carbide-looking precipitates can be found. The secondary dendrite arm spacings (SDASs, λ_2_) were measured (Table 2) and the results indicate that the pouring temperature had a stronger effect on the obtained values. The average distance in the castings was in the range of 63–66 μm, 69–72 μm, and 72–77 μm for 0 wt%, 5 wt%, and 10 wt% of CoAl_2_O_4_, respectively. According to the equation for λ_2_~(t_s_)^1/3^~(1/G_v_)^1/3^, the temperature gradient (G), local solidification time (t_s_), and cooling rate (v) influence the SDAS [26]. Values strictly depend on the casting parameters (e.g., shell mold thickness, preheating and pouring temperature, cooling rate after solidification) and heat transfer between the nickel-based superalloy and the ceramic shell mold, originating from their thermophysical features. In general, the investment casting of Ni-based superalloys is characterized by low cooling rates due to the very low thermal conductivity of the shell molds. Matysiak [27] suggested that the cooling rate of the Inconel 713C^®^ nickel-based superalloy at 1263–650 °C after casting is approx. 10–12 °C/min. The thermal conductivity of the shell mold, which consisted of a zircon filler, colloidal silica binder, and alumina silicate powders as a backup material, was 1.09 W/(m·K) at 1200 °C and 0.77 W/(m·K) at 600 °C [27].

**Figure 5:**
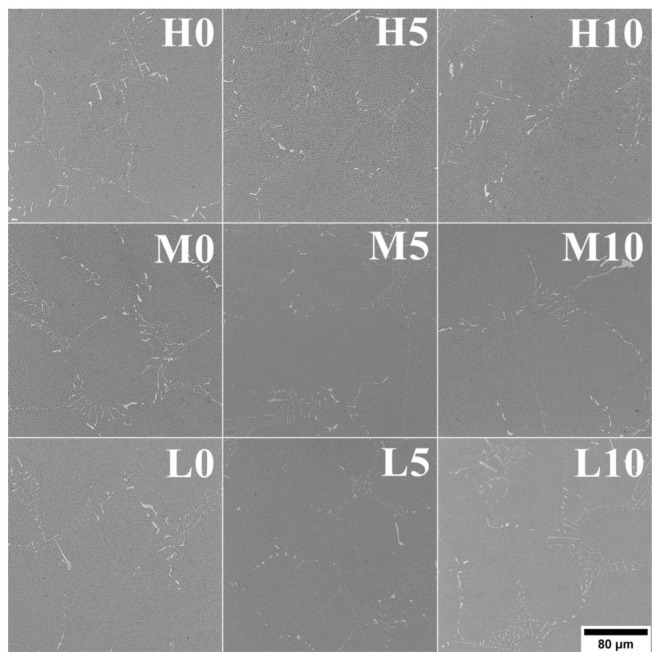
The distribution, morphology, and size of MC carbides in the as-cast Inconel 713C^®^ castings, SEM-BSE.

Based on ThermoCalc^®^ simulations and microstructural characteristics, it can be stated that the formation of the γ matrix phase occurs in the initial solidification stage. As the temperature decreases, the solubility of strong carbide-formers, such as Nb, Ti, and C, in the formed matrix decreases, leading to their segregation at the interface between the matrix dendrite and the interdendritic liquid [24]. Notably, k^i^ = 1 indicates a uniform distribution of alloying elements. These conditions promote carbide precipitation, which can appear in various forms ranging from blocky shapes to Chinese script-like structures, as shown in Figure 5. Bhambri [28] observed that carbide morphology transforms with increasing cooling rates. Initially, carbides appear octahedron-shaped, resembling blocks in a two-dimensional image. In this work, carbide growth conditions were closer to an equilibrium state, with slower cooling rates. However, with higher cooling rates, the shape of the carbides changed to a more arrow-like structure and eventually evolved into shapes reminiscent of Chinese script. At these higher cooling rates, the carbide growth rate increased, accompanied by the sufficient diffusion of carbide-forming elements. The formation of equilibrium shapes becomes increasingly challenging, being affected by the heat flow direction and the distribution of carbide-formers.

**Figure 6:**
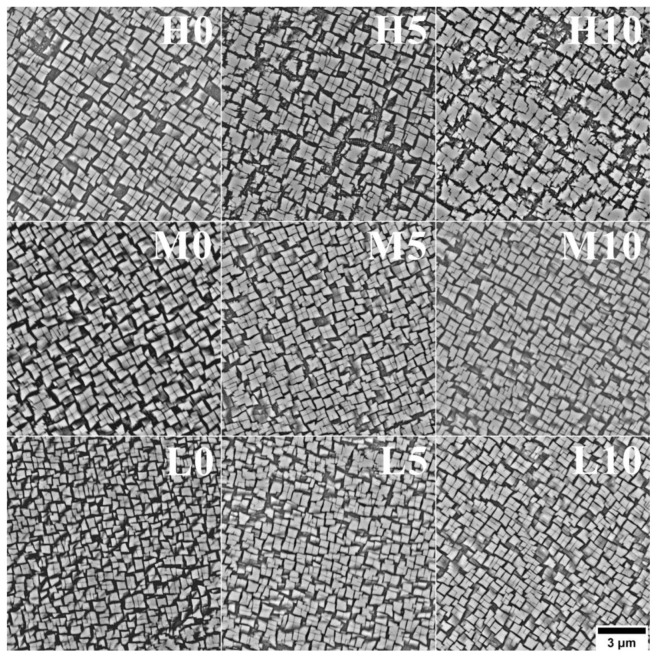
Morphology of secondary γ’ precipitates in dendritic regions in the as-cast Inconel 713C^®^ castings, SEM-BSE.

**Figure 7:**
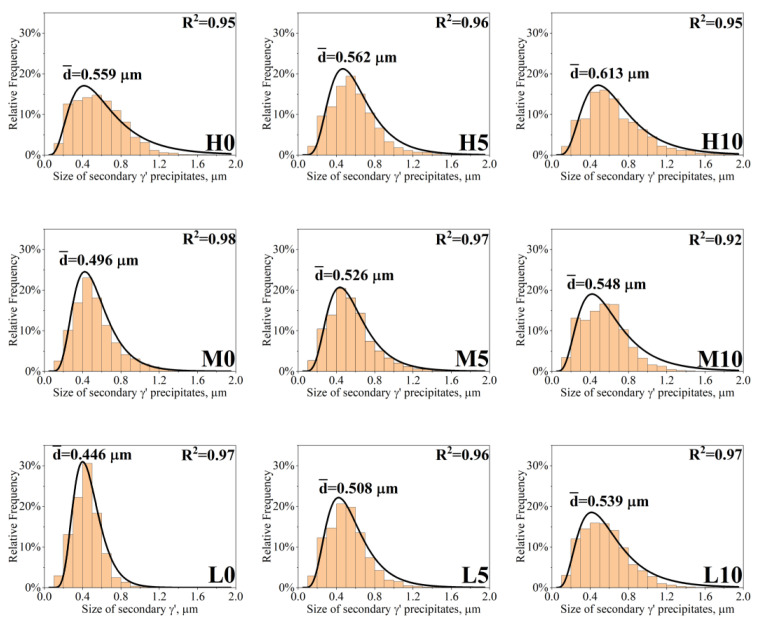
Size of secondary γ’ precipitates in dendritic regions in the as-cast Inconel 713C^®^ castings.

The morphology of the secondary γ’ precipitates in the dendritic regions is close to cubic (Figure 6). The local thermodynamic conditions influencing the morphology are determined by the chemical composition, state of elastic stresses, and the mutual interaction between precipitates [29]. These factors change as a function of temperature and are conditioned by the system’s attempt to achieve thermodynamic equilibrium. The mean size of secondary γ’ precipitates expressed as the equivalent side of the square was calculated to quantitatively analyze the differences originating from the various melt-pouring temperatures and inoculant contents (Figure 7). For castings produced within the unmodified shell mold, the mean size of secondary γ’ precipitates increased from 0.446 μm to 0.559 μm with increasing pouring temperature. With the addition of inoculant to the primary coating, the mean size of secondary γ’ precipitates tended to be greater. For T_0_ = 1450 °C, the mean size was 0.508 μm and 0.539 μm for 5 wt% and 10 wt% CoAl_2_O_4_, respectively; for T_0_ = 1480 °C, it was 0.526 μm and 0.548 μm; and for T_0_ = 1520 °C, it was 0.562 μm and 0.613 μm. Sims [2] observed that slower cooling rates during the directional solidification process could result in a coarser solid-state cuboidal γ’ directly in the as-cast state, which is in line with our observations. The higher preheating mold temperature caused a decrease in casting cooling, giving more time for nuclei to re-precipitate and for the γ′ nucleus to grow. The influence of the γ’ phase’s size on superalloys’ strength is complex, especially since they have various chemical compositions and most are subjected to heat treatment [30]. Simultaneously, Inconel 713C^®^ can be serviced without such treatment. However, there is a possibility of having precipitates finer than the critical value, meaning that they are small enough for dislocations to cut through them. This critical mean size can range depending on the superalloy type. If this value is crossed, the Orowan mechanism occurs [31]. Additionally, γ’ precipitates have an ordered L1_2_ crystal structure, so dislocations can cut or bypass them in various complex forms, depending on the stress and service temperature [32]. Nathal [33] observed in single-crystal nickel-base superalloys that the cube-like morphology exhibits the best creep properties among all morphologies, while the optimal initial size of the precipitates is around 0.50 μm.

**Figure 8:**
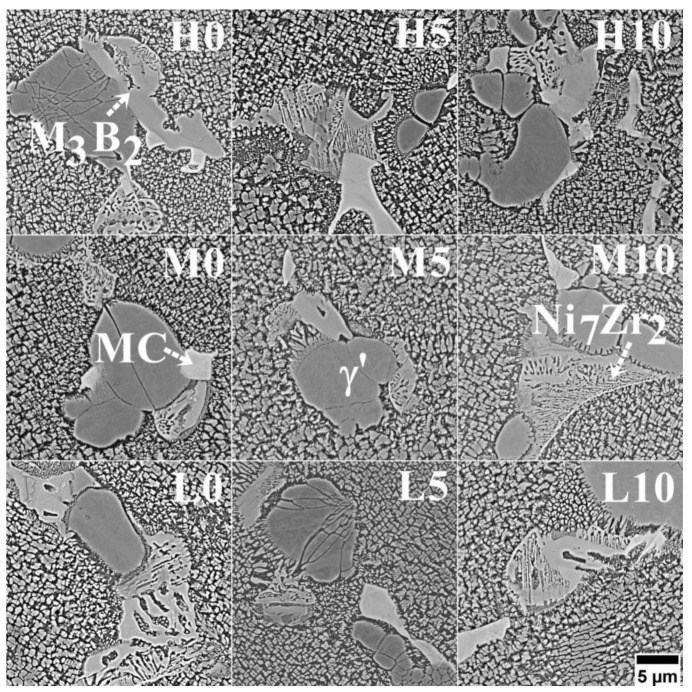
Morphology of the precipitates in the interdendritic spaces of as-cast Inconel 713C^®^ castings, SEM-BSE.

**Figure 9:**
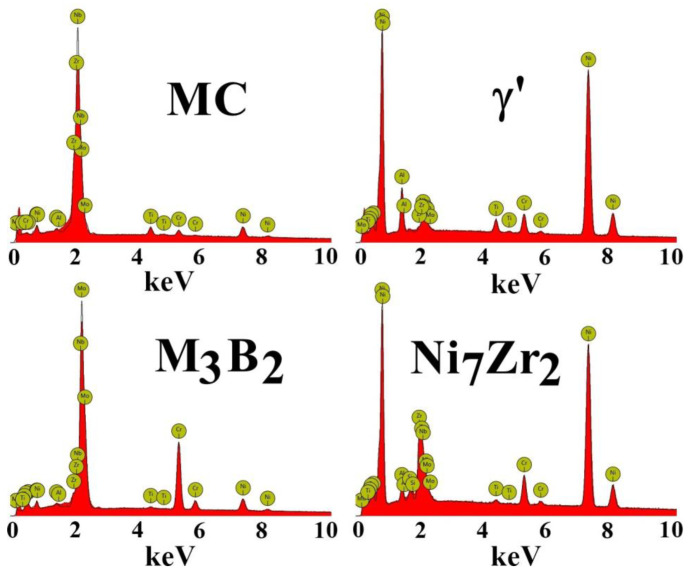
The EDX spectra of the phases detected in as-cast Inconel 713C^®^ castings, SEM-EDX.

Numerous phases formed in the castings’ interdendritic spaces due to alloying element segregation during solidification. Based on SEM-EDX, it was possible to determine that the same phases are present in all castings, regardless of the selected pouring temperature or inoculant content. Morphology images and corresponding qualitative spectra are presented in Figure 8 and Figure 9, respectively. A diversified distribution and complex morphology characterize primary γ’ precipitates, which formed via the L→γ + γ’ eutectic phase transformation. Eutectic γ-γ’ indicates a strong Al enrichment in the residual liquid during casting solidification. The γ-γ’ is an undesirable constituent, and heat treatment can lead to its decrease. Its amount is relatively low compared to that in other superalloys with similarly high Al concentrations, like MAR-M247 [34] or Renè 108 [35]. In the near-eutectic, the carbides are significantly enriched in Nb, Mo, and Ti.

The SEM-EDX analysis of the gray-phase contrast precipitate revealed an increased concentration of Mo and Cr (Figure 8 and Figure 9). The size of some of these precipitates locally exceeded 5–10 μm. Similar findings were obtained for various Ni-based superalloys, indicating the presence of borides, such as M_3_B_2_ and M_5_B_3_ [36]. M_5_B_3_ borides are strongly enriched in W and Cr, while M_3_B_2_ borides are strongly enriched in Mo and Cr. Since Inconel 713C^®^ lacks tungsten, M_5_B_3_ is not expected to be present, and only M_3_B_2_ is likely to form (which was also confirmed by ThermoCalc^®^ simulations). Boron is added to Ni-based superalloys to increase creep strength, and its nominal concentration in Inconel 713C^®^ is 0.012 wt%. Considering borides’ very low solubility in γ and γ’, favorable conditions for their formation exist only at the end of solidification. The presence of such large boride precipitates indicates that the solution heat treatment temperature should be selected carefully so as not to lead to the local melting of B-rich areas. The relationship between size, distribution, boride fraction, and melt-pouring temperature or inoculant content was not observed due to their irregular presence in the interdendritic spaces. Apart from near-eutectic γ-γ’ islands and borides, precipitates with a lamellar-like morphology are also observed. SEM-EDX spectra revealed an increased concentration of Ni and Zr. Matysiak [37] observed with selected area electron diffraction (SAED) that these precipitates are Ni_7_Zr_2_. On the Ni-Zr binary diagram, the Ni_7_Zr_2_ phase (mC36, C2/m (12)) precipitates at 1438 °C through a congruent phase transformation, L → Ni_7_Zr_2_ [38]. Subsequently, two peritectic transformations occur: at 1304 °C, L + Ni_7_Zr_2_ → Ni_5_Zr (cF24, F-43m), and at 1181 °C, L + Ni_7_Zr_2_ → Ni_21_Zr_8_ (aP29, P-1). Only one eutectic transformation resulting in the formation of the γ phase (FCC), namely L → γ + Ni_5_Zr at 1164 °C, takes place. Consequently, the Ni_7_Zr_2_ phase can be present in Inconel 713C^®^ but not in the form of a γ-Ni_7_Zr_2_ eutectic, indicating that its formation is likely more complex, as suggested by the morphology of the precipitates. During the final stage of solidification, the residual liquid in the nickel-based superalloy becomes enriched in Zr due to a peritectic transformation with the eutectic phase or primary γ’ phase, namely L + γ’ → γ + Ni_7_Zr_2_ [39]. The superalloy’s complex chemical composition allows for a ternary eutectic transformation at the end of casting solidification. One of the ternary eutectic transformations leading to Ni_7_Zr_2_ formation is L → γ + Ni_7_Zr_2_ + Ni_5_Zr. However, the presence of Ni_5_Zr was not detected in Inconel 713C^®^ by other authors [40,41]. Similar findings confirming the Ni_7_Zr_2_ phase have been reported for Inconel 939^®^ [42] and Inconel 738^®^ [43]. The morphology and microanalysis of carbides (MC-type) located near the Ni_7_Zr_2_ compound reveal a strong enrichment in Nb and Zr, which can indicate that during casting solidification, a ternary eutectic transformation occurs: L (residual with high Zr and C content) → γ + Ni_7_Zr_2_ + (Nb, Zr)C. Babu [44] suggested that eutectic-type transformations in chemically complex nickel-based superalloys can be more intricate. The enrichment of solutes in the residual solidifying liquid may be insufficient to initiate a typical eutectic transformation, primarily due to the minimal volume fraction of eutectic γ-γ’. Microstructure investigations show that the M_3_B_2_ is adjacent to Ni_7_Zr_2_ and eutectic γ-γ’ islands. These multiphase regions indicate that the formation of eutectic γ-γ’ islands can coincide with the precipitation of a ternary eutectic from the remaining liquid, following the sequence L (enriched in Zr and B) → γ + Ni_7_Zr_2_ + M_3_B_2_. The observed morphology of constituents in our Inconel 713C^®^ castings aligns with Babu’s interpretation [44], possibly explaining the formation of primary phases in the interdendritic regions. According to Murata [45], the intermetallic phase Ni_7_(Hf, Zr)_2_ is not stable at the selected intermediate temperature (like heat treatment, creep, or service). It undergoes the phase transformation MC + (Ni, Co)_7_(Hf, Zr)_2_ + Cr (in the matrix) → (Hf, Zr)C + Cr_23_C_6_ + γ. It should be noted that the Ni_7_Hf_2_ and Ni_7_Zr_2_ phases have the same crystallographic structure and can dissolve each other and have similar behavior during thermal exposure.

### 3.3. Mechanical Properties of Inconel 713C^®^ Castings at Room and Elevated Temperature

Tensile test results are presented in Table 3. The Inconel 713C^®^ superalloy, when poured at 1450 °C, exhibits an average yield strength of 787 MPa. However, when cobalt aluminate is added to the first layer of the ceramic mold, the YS increases to 813 MPa and 835 MPa for 5 wt% and 10 wt%, respectively. At a melt-pouring temperature of 1480 °C, the unmodified casting had an average YS of 783 MPa, while for the modified casting, the measured values were at least 27 MPa higher. When T_0_ = 1520 °C, the unmodified castings exhibit a YS of 775 MPa. With the addition of CoAl_2_O_4_, the YS increased to 804 MPa (5 wt%) and 819 MPa (10 wt%). These results demonstrate that the yield strength of the unmodified castings remained below 800 MPa for each pouring temperature. Castings L0 (1450-0) and M0 (1480-0) exhibited the highest standard deviation in YS. However, the average YS values exceed 800 MPa when cobalt aluminate was included in the first layer of the ceramic mold. Regarding UTS, the values did not exceed 900 MPa for the unmodified castings. However, selecting the optimal pouring temperature and CoAl_2_O_4_ concentration allowed achieving values exceeding 1000 MPa. These results suggest that adding cobalt aluminate in the first layer has a significantly greater impact on the YS and UTS in comparison to pouring temperature changes. This effect can be attributed to the role of CoAl_2_O_4_, which is aimed at grain refinement. Following the AMS5291 standard [22], the minimum YS of Inconel 713C^®^ castings should be higher than 690 MPa, while UTS should be more than 758 MPa, which is in line with what was achieved in this study.

**Figure 10:**
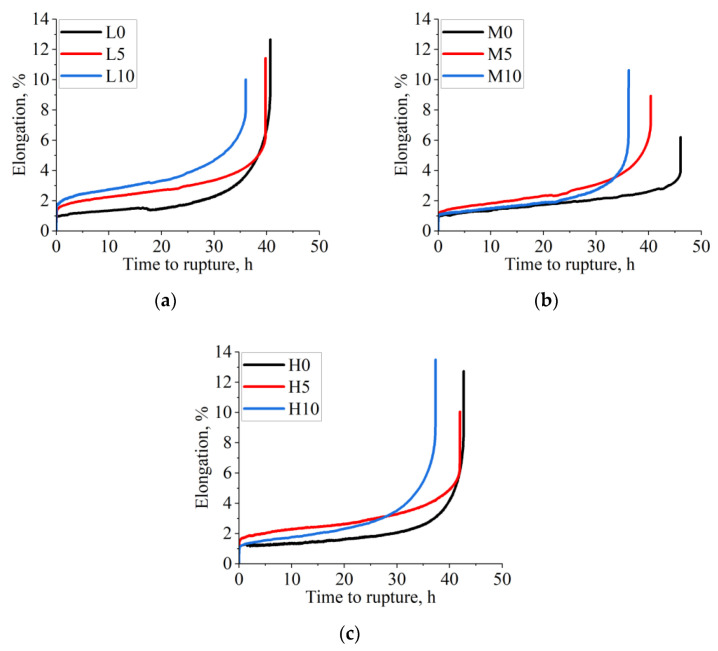
Creep curves of as-cast Inconel 713C^®^ castings: (**a**) L0-L10; (**b**) M0-M10; (**c**) H0-H10.

Figure 10 presents the creep curves of the as-cast Inconel 713C^®^ castings (982 °C/152 MPa), with one representative curve selected for each variant. The recorded curves consist of three characteristic stages. Stage I corresponds to the initial deformation, where the creep rate is the highest, followed by stage II, i.e., steady-state creep, and stage III, where the sample is fractured. The sample poured at 1450 °C into the mold without CoAl_2_O_4_ exhibited a time to rupture of approx. 40.7 h. However, with the addition of the modifier, the time to rupture dropped to 39.8 h for 5 wt% CoAl_2_O_4_ and 36.0 h for 10 wt% CoAl_2_O_4_. At the pouring temperature of 1480 °C, the unmodified casting had a time to rupture of about 46.1 h. The presence of cobalt aluminate in the amount of 5 wt% or 10 wt% reduced this time to 40.4 h and 36.2 h, respectively. For the highest tested pouring temperature, the unmodified superalloy sample’s time to rupture was 42.7 h, whereas for the modified sample, it was 40.2 h for 5 wt% addition and 37.3 h for 10 wt%. Notably, the casting with the longest time to rupture also exhibited the lowest steady-state creep rate. This characteristic is highly beneficial during service, as it indicates improved resistance to deformation over time. Under typical stresses in service, the steady-state creep rate decreases with increasing grain size, while YS and UTS usually decrease, which is visible in the performed experiments. According to the AMS5391 standard [23], the expected minimum time to rupture is 30 h under the assumed test parameters, which states that the selected manufacturing parameters and composition of the primary coat in the shell mold guarantee sufficient creep properties.

**Figure 11:**
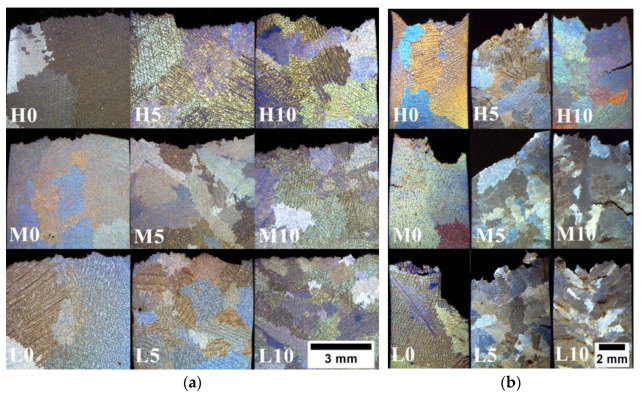
Microstructure of the cross-sectioned specimens after (**a**) tensile tests and (**b**) stress-rupture tests.

Figure 11a displays the microstructures of cross-sectioned specimens following the tensile test, revealing the nature of cracking and their development along the grain boundaries. The grain size influences the strength primarily due to the different crystallographic orientations present in adjacent grains. Grain boundaries play a crucial role in impeding the movement of dislocations, which can be attributed to two main factors [46]. Firstly, when a dislocation transitions from one grain to another, it encounters a change in its direction of motion due to the distinct orientations of the two equiaxed grains. This alteration in direction acts as a barrier to dislocation movement. Secondly, within the region of a grain boundary, atomic disorder exists, resulting in a discontinuity of slip planes from one grain to the next one. This discontinuity further obstructs the easy progression of dislocations between adjacent grains. Figure 11b illustrates the microstructure of crept Inconel 713C^®^, revealing the presence of intergranular cracks along with numerous secondary cracks and local voids. Creep mechanisms can be categorized into two groups: those influenced by grain size and those not [47]. In both cases, the creep rate is connected to the diffusion rate. The process of diffusion creep, involving the creation and disappearance of vacancies at boundaries, is highly dependent on grain size. Dislocation creep occurs within the grains and is not influenced by grain size. When subjected to constant stress and temperature, an increase in grain size leads to a significant decrease in the contribution of dislocation creep. Simultaneously, the rate of diffusion creep also decreases. When the homologous temperature (ratio of test temperature and solidus temperature) exceeds 0.7, the dominant creep mechanism is Nabarro–Herring creep [48]. This type of creep occurs as vacancies migrate within grains, moving from regions under tension to compressed regions and through volume diffusion in the opposite direction. In contrast, at homologous temperatures ranging from 0.4 to 0.7, Coble creep becomes more prevalent, which operates through diffusion along grain boundaries [49]. This means that the creep rate via the Nabarro–Herring mechanism is proportional to 1/d^2^ (d—grain size), while Coble creep is proportional to 1/d^3^. Coble creep is more strongly influenced by grain size compared to Nabarro–Herring creep. As a result, the contribution of Coble creep is considered negligible in fine-grained materials. Consequently, coarse-grained materials exhibit higher resistance to creep.

Apart from grain size, the mechanical properties are influenced by a high volume fraction of γ’ precipitates and the presence of other strengthening phases, narrow γ matrix channels, and the strengthening effect of solid solution in both the matrix and γ’ phases. The total content of γ’-formers in the material exceeds 6 wt%, which is relatively high compared to that in other superalloys commonly used in aerospace engines [2]. The γ’ phase undergoes deformation primarily through slip-on systems characterized by {111} <110> planes. Dislocations in the γ’ phase have a Burger vector with a length of a√2, which is twice as long as that in the matrix. When a dislocation in the γ’ precipitate slips by a distance equal to the length of the Burger vector, it disrupts the ordering of the crystal lattice. This disruption leads to the creation of interfaces, which are defects characterized by high energy. The dislocation movement within γ’ precipitates becomes significantly more challenging due to these high-energy interface defects [50,51,52].

## 4. Grain Refinement Mechanism via CoAl_2_O_4_ Inoculant Incorporation

A fine-grained and more uniform structure can be obtained via the chemical reaction of the inoculant, included in the primary coating of the shell mold, with selected alloying elements in the superalloy [53,54]. Feagin [55] indicated that the use of more stable CoAl_2_O_4_ or Co_2_SiO_4_ compounds as the nucleus does not exclude the possibility of their decomposition into, among others, pure nanoparticles of Co when in contact with a reducing atmosphere or the liquid alloy. To investigate the mechanism of Co particle formation and morphology during casting, Jian [20] examined several shell molds with CoAl_2_O_4_ as the primary layer. Upon observing the molds after contact with a liquid superalloy, they discovered three distinct layers: white, blue, and black (corresponding to the colder to hotter sections). The unmelted portion of the molds, known as the backup part, consisted of Al_2_O_3_ and SiO_2_ in cristobalite and an amorphous phase. The transitional region (blue) was predominantly composed of cobalt aluminate, amorphous silicate, and fine metallic cobalt particles measuring up to a maximum diameter of 4 μm. The amount of amorphous phase and metallic cobalt steadily increased as the shell mold became hotter. In the final black region (0.02 mm) of the primary layer, the major constituents were Co, Al_2_O_3_ (some of which formed solid solutions with chromium or cobalt), and a small quantity of cristobalite. These constituents also exhibited a gradual change. Notably, the number of cobalt particles in the black layer exceeded the amount in the blue layer. CoAl_2_O_4_ and silicates transformed into mineral phases primarily composed of Al_2_O_3_ solid-solution, with Cr and Co as solutes. To determine which of the elements could reduce cobalt aluminate to its metallic form and identify the remaining products, an experiment involving the annealing of metal powders (Al, Cr, Ti) with CoAl_2_O_4_ was conducted. The mixtures were subjected to heat treatment at 1200 °C for 2 h under vacuum and subsequently analyzed using X-ray diffraction. During the pouring of liquid metal into the shell mold cavity, the CoAl_2_O_4_ present in the primary layer underwent reduction. As a result, pure Co particles were formed according to the following reactions (Equations (1)–(3)):

CoAl_2_O_4_ + 2/3Al → 3/4 Al_2_O_3_ + Co + q (1)

CoAl_2_O_4_ + 2/3 Cr → 1/3 Cr_2_O_3_ + Co + Al_2_O_3_ + q (2)

CoAl_2_O_4_ + 1/2 Ti → 1/2 TiO_2_ + Co + Al_2_O_3_ + q (3)

The reduction capacities of the selected metals vary. The highest amount of obtained Co particles occurred after the reaction with Al and then decreased in sequence when reacting with Cr and Ti. The residual amount of cobalt aluminate increased in the same order. Reactions with Al and Cr allowed for decomposing 95 wt% and 70 wt% of cobalt aluminate, respectively. Crystallographic matching at the inoculant–matrix interface has typically served as a good indicator for the potency of nucleating substrates in terms of the low ΔT_n_ (nucleation undercooling) required to nucleate grains, and the formation of solid Co particles is ideal as they possess an FCC lattice structure with a small lattice mismatch with the γ phase. From microstructural observations of the Inconel 713C^®^ castings and described experiments conducted by Jian [20], it can be concluded that during the melt-pouring process, the cobalt aluminate compound reacted with Al, Cr, and Ti alloying elements (total concentration exceeds 20 wt%). Grain refinement occurred through the increase in the heterogeneous nucleation rate.

## 5. Conclusions

In this work, the influence of melt-pouring temperature (L—low; M—medium, H—high) and the concentration of CoAl_2_O_4_ (0 wt%, 5 wt%, 10 wt%) in the primary coating of the shell mold on Inconel 713C^®^ castings’ grain size, microstructure, and strength at room temperature and 982 °C was shown. The main conclusions are as follows:

1. Grain size control in lost-wax Inconel 713C^®^ castings can be performed by changing the melt-pouring temperature and CoAl_2_O_4_ contents in the prime coating of the shell mold. The most significant grain refinement was achieved in casting H10, where the grain size was reduced from 6.55 mm to 2.36 mm.
2. The melt-pouring temperature had a greater influence on the SDAS than the inoculant content. The lowest average SDAS (63 μm) was achieved in the L0 casting, whereas the highest was achieved in the H10 casting (77 μm).
3. The size of secondary γ’ in the dendritic regions exhibited a log-normal distribution with increasing melt-pouring temperature, whereas the mean size increased with increasing inoculant contents. The finest mean precipitate size was in casting L0 (0.446 μm), while the coarsest was in casting H10 (0.613 μm).
4. Primary and secondary γ’, MC carbides, M_3_B_2_ borides, and the intermetallic Ni_7_Zr_2_ phase were found in the interdendritic regions of all castings, regardless of the applied melt-pouring temperature or CoAl_2_O_4_ concentration.
5. Grain refinement influenced the mechanical properties of the Inconel 713C^®^ superalloy at ambient and elevated temperatures. In unmodified variants, the average YS decreased with increasing melt-pouring temperature from 787 MPa (L0–1450 °C) to 775 MPa (H0–1520 °C). Among the CoAl_2_O_4_-modified variants, the highest average YS of 835 MPa was achieved in casting L10.
6. With increasing inoculant content and, in turn, grain size refinement, the creep resistance tended to be lower, while all samples exceeded the required minimum time to rupture of 30 h (max. 46.1 h for casting M0).

## Figures and Tables

**Table 1:** Technological parameters used to prepare the Inconel 713C^®^ castings.

Casting	Pouring Temperature, °C	Inoculant Content, wt%	Shell Mold Temperature, °C
L0	1450	0	1150
L5		5	
L10		10	
M0	1480	0	
M5		5	
M10		10	
H0	1520	0	
H5		5	
H10		10	

**Table 2:** Mean grain size of Inconel 713C^®^ castings.

Pouring Temperature, °C	CoAl_2_O_4_, wt%	Grain Size, mm	SDAS, μm
1450	0	4.40 (±3.56)	63 (±7)
	5	3.77 (±4.75)	63 (±5)
	10	2.44 (±2.41)	66 (±4)
1480	0	3.18 (±3.34)	69 (±5)
	5	2.68 (±2.57)	69 (±5)
	10	2.40 (±2.07)	72 (±5)
1520	0	6.55 (±3.73)	72 (±8)
	5	2.45 (±2.62)	77 (±7)
	10	2.36 (±2.32)	77 (±6)

**Table 3:** Tensile strength of as-cast Inconel 713C^®^ castings.

Pouring Temperature, °C	CoAl_2_O_4_, wt%	YS, MPa	UTS, MPa
1450	0	787 (±21)	879 (±14)
	5	813 (±2)	1010 (±7)
	10	835 (±6)	994 (±28)
1480	0	783 (±23)	877 (±5)
	5	828 (±1)	984 (±6)
	10	814 (±2)	970 (±21)
1520	0	775 (±8)	868 (±18)
	5	804 (±14)	947 (±46)
	10	819 (±8)	967 (±14)

## Data Availability

Not applicable.
